# Retrieval of an Undeflatable Balloon During Percutaneous Coronary Intervention in Combination With Small Thoracotomy

**DOI:** 10.1016/j.jaccas.2025.105157

**Published:** 2025-09-24

**Authors:** Koya Okabe, Kenichi Hagiya, Itaru Takamisawa, Tomohiro Iwakura, Mamoru Nanasato

**Affiliations:** a Department of Cardiology, Sakakibara Heart Institute, Tokyo, Japan; b Department of Cardiovascular Surgery, Sakakibara Heart Institute, Tokyo, Japan

**Keywords:** balloon retrieval, coronary complications, percutaneous coronary intervention, small thoracotomy, undeflatable balloon

## Abstract

**Background:**

An undeflatable balloon is a rare but potentially life-threatening complication of percutaneous coronary intervention (PCI).

**Case Summary:**

An 80-year-old man undergoing PCI developed an undeflatable balloon lodged in the proximal left anterior descending artery. Catheter-based retrieval attempts were unsuccessful, necessitating surgical intervention. The balloon was successfully deflated via a small thoracotomy through direct puncture with a 25-gauge needle.

**Discussion:**

This case demonstrates the successful surgical retrieval of an undeflatable balloon using a minimally invasive off-pump approach. To our knowledge, this is the first reported case of successful retrieval of an undeflatable coronary balloon by direct needle puncture through a small thoracotomy without a coronary artery incision.

**Take-Home Messages:**

Undeflatable balloons are a potentially fatal complication during PCI. If catheter-based retrieval fails, surgical intervention becomes necessary. A minimally invasive approach, such as off-pump small thoracotomy with direct coronary puncture, may be preferable, particularly in patients at high surgical risk.

## Visual Summary

**Figure:**
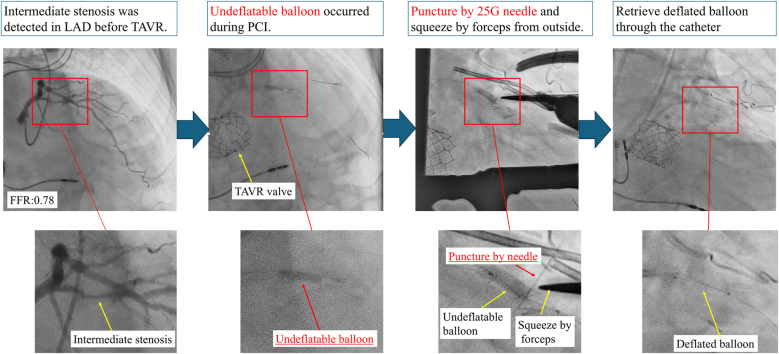

**Visual Summary:**
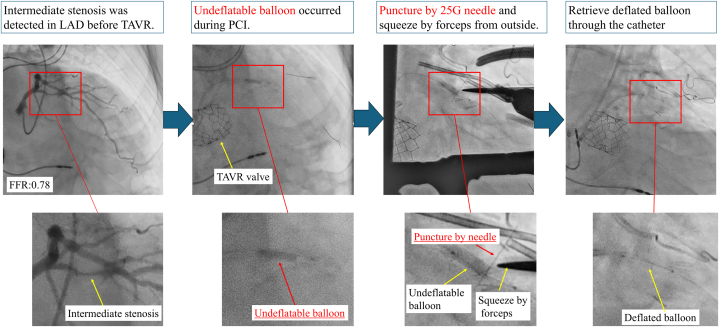
Process of Surgical Retrieval for an Undeflatable Balloon Illustration shows retrieval of an undeflatable coronary balloon via small thoracotomy and direct coronary puncture using a 25-G needle. FFR = fractional flow reserve; LAD = left anterior descending artery; PCI = percutaneous coronary intervention; TAVR = transcatheter aortic valve replacement.

## History of Presentation

**Figure 1:**
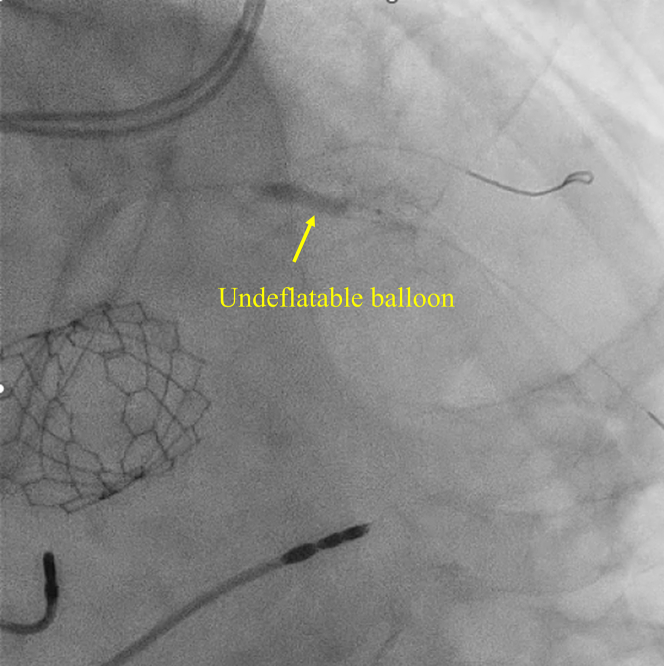
Undeflatable Balloon in Coronary Artery Coronary angiography demonstrating an undeflatable balloon lodged in the proximal left anterior descending artery.

An 80-year-old man presented with dyspnea and was diagnosed with severe aortic stenosis. The patient subsequently underwent transcatheter aortic valve replacement (TAVR). Preoperative coronary angiography revealed intermediate stenosis in the proximal left anterior descending artery (LAD) (Supplemental Figure 1). Positive findings on resting full-cycle ratio (0.82) and fractional flow reserve (0.78) prompted the decision to perform percutaneous coronary intervention (PCI). PCI was performed 4 months after TAVR. A 6-F sheath was inserted into the right radial artery. A Judkins Left 4.0 guiding catheter and a guide extension catheter were used to access the LAD. A 0.014-in floppy guidewire crossed the lesion, and intravascular ultrasonography confirmed appropriate wire placement. A drug-eluting stent (3.0 × 33 mm) was deployed after predilation. Postdilation with a noncompliant balloon (3.5 × 15 mm) was performed at 12 atm; however, the balloon failed to deflate and became lodged in the proximal LAD (Figure 1). The inflator contained a 1:1 mixture of contrast agent and normal saline.

**Take-Home Messages**

• Undeflatable balloons are a potentially fatal complication during PCI; if catheter-based retrieval fails, surgical intervention becomes necessary.
• A minimally invasive approach, such as off-pump small thoracotomy with direct coronary puncture, may be preferable, particularly in patients at high surgical risk.

## Past Medical History

The patient had a history of sick sinus syndrome treated with a pacemaker and had undergone TAVR 4 months earlier.

## Differential Diagnosis

Mechanical balloon failure and insufflator malfunction were considered the primary causes of the failure to deflate.

## Investigations

Negative pressure was applied using both an insufflator and syringe, but the balloon could not be deflated. Forceful traction resulted in the guiding catheter being drawn inward; however, the balloon remained lodged (Video 1).

## Management

Although ST-segment elevation was observed in the precordial leads, the patient's symptoms were mild and blood pressure remained stable. Catheter-based retrieval was initially attempted. A 5-F straight guiding catheter with a cut tip was advanced, but could not reach the balloon because it was caught in the stent strut. A 4-F straight guiding catheter with a cut tip reached the proximal edge of the balloon; however, attempts to rupture the balloon failed. As ST-segment elevation persisted, an intra-aortic balloon pump was inserted through the left femoral artery. A guide extension catheter with the tip cut off was used; however, pressing it against the balloon failed to rupture. Next, a 7-F sheath was placed in the right femoral artery for double-guide access. Multiple guidewires (tip loads of 0.6, 0.8, and 1.0 g) were used, but none could pass through the balloon. A guidewire designed for chronic total occlusion (tip load, 12 g) was used to perforate the balloon, but it slipped and failed.

**Figure 2:**
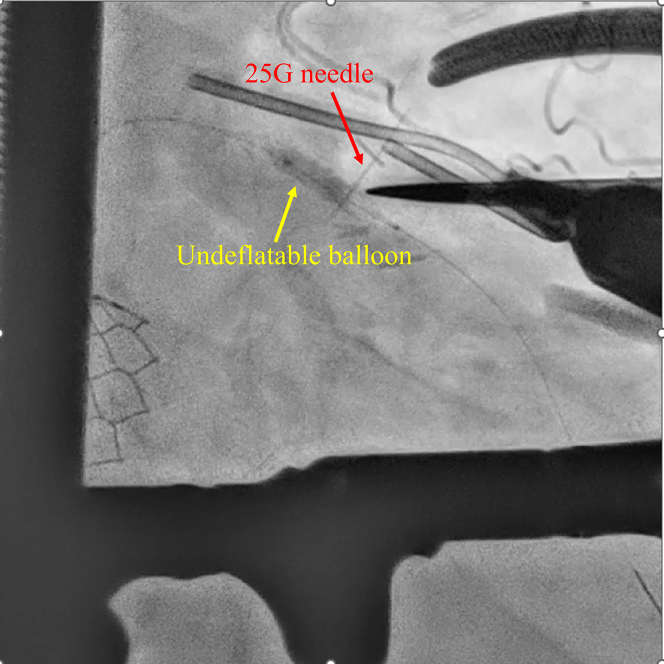
Needle Puncture of an Undeflatable Balloon A 25-G needle was used to puncture the undeflatable balloon from the coronary artery surface.

**Figure 3:**
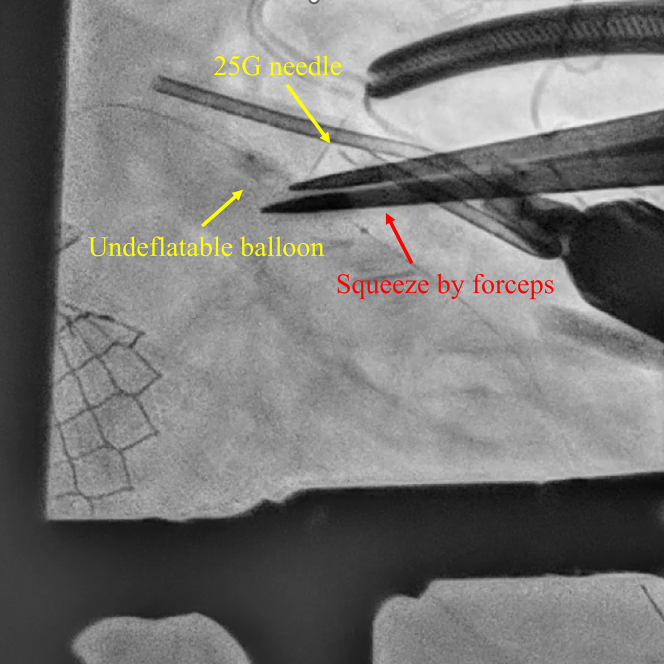
Squeezing the Punctured Balloon Using Forceps After repeated punctures, the balloon was manually squeezed with forceps, leading to successful deflation.

**Figure 4:**
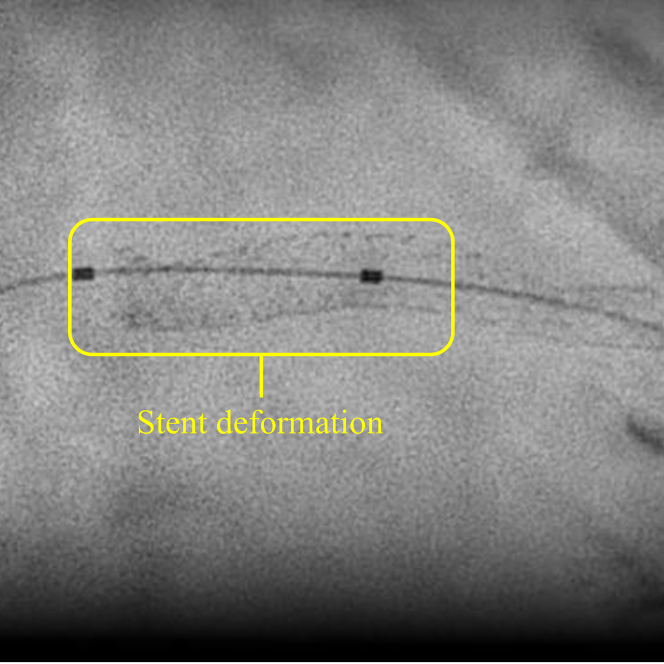
Stent Deformation After Retrieval of the Balloon Magnified angiographic image showing focal deformation of the stent structure in the proximal to mid-left anterior descending artery.

Given persistent ST-segment elevation, further catheter-based retrieval was deemed unfeasible, and surgical retrieval was performed. Under general anesthesia, the heart was exposed via a small 3-cm left anterior thoracotomy. The LAD was directly visualized, and fluoroscopy was used to identify the position of the balloon. A 25-G tuberculin needle was used to puncture the balloon externally (Figure 2, Video 2). Despite multiple punctures, the balloon did not deflate. However, direct compression of the coronary segment with forceps facilitated balloon deflation (Figure 3, Video 3). The total time from balloon entrapment to successful deflation was 194 minutes. The balloon was then retrieved using a guiding catheter (Video 4). Because angiography confirmed deformation in the proximal to midsite of the stent (Figure 4), the stent was dilated using a semicompliant balloon (3.5 × 15 mm) for the proximal site and a noncompliant balloon (3.0 × 6 mm) for the midsite. No bleeding was observed at the LAD artery puncture site (Video 5).

## Outcome and Follow-Up

The thoracotomy was closed, and the patient was transferred to the intensive care unit with an intra-aortic balloon pump in place. Postoperatively, creatine kinase and creatine kinase-muscle/brain isoenzyme levels increased to 6,625 and 344.1 U/L, respectively. The patient developed cardiogenic shock, requiring catecholamines and fluid resuscitation. Subsequently, the patient experienced acute heart failure, which required intensive medical therapy.

Intensive care was continued, and the intra-aortic balloon pump was removed on postoperative day 4. The patient was discharged from the intensive care unit on day 14 and hospital on day 32. One year postoperatively, the patient remained hospitalization-free because of heart failure or revascularization.

## Discussion

PCI is widely used to treat coronary artery disease; however, complications such as an undeflatable balloon can occur. Although rare, an undeflatable balloon is a serious complication that may lead to coronary artery occlusion and has life-threatening consequences. The patient underwent successful treatment through surgical methods involving a small thoracotomy after unsuccessful interventional approaches.

During the procedure, the same indeflator and contrast media were used for all balloons, ruling out crystallized contrast as a possible cause. The balloon in question was used for the first time during poststent dilatation and advanced smoothly into the stent, making overstretching of the balloon shaft unlikely. Although the exact mechanism of deflation failure could not be confirmed, most cases of undeflatable balloons are attributed to mechanical obstruction of the balloon shaft, which prevents deflation.^1^^,^^2^ Various cases and retrieval methods using catheters have been reported to date.^2^, ^3^, ^4^, ^5^ Possible causes include balloon shaft deformation or internal leakage, which may impair the negative suction required for deflation.

In this case, bailout of the undeflatable balloon was performed in accordance with a clinical expert consensus document on bailout algorithms for complications in PCI^6^; however, catheter-based retrieval was unsuccessful. Two key factors may explain the previously reported failure of catheter-based retrieval methods. First, an undeflatable balloon was placed after stent implantation. This complication occurs when attempting to further compress an insufficiently expanded area of the stent, leading to inadequate stent apposition to the vessel wall. Consequently, significant difficulties have been encountered in delivering various retrieval tools. Second, complications occurred after TAVR valve implantation. PCI after TAVR is more challenging because of anatomic changes in the aortic valve complex and interference from the TAVR valve.^7^ In this case, PCI was performed under conditions where the guiding catheter could not adequately engage the coronary artery, which likely contributed to the difficulty of catheter-based retrieval.

Surgical retrieval of an undeflatable balloon should be considered when catheter-based retrieval fails. Surgical retrieval is rarely required, with only 2 prior cases reported.^8^^,^^9^ In both cases, the balloon shaft was torn during catheter-based retrieval, necessitating a coronary artery incision after the establishment of a cardiopulmonary bypass to retrieve an undeflatable balloon. There are 2 key differences between our case and the previously reported cases. First, although previous cases involved on-pump surgery, our case was managed using an off-pump procedure. Off-pump surgery has been demonstrated to be less invasive compared with on-pump surgery, making it a preferred approach, particularly in patients with high surgical risk.^10^ Second, our retrieval method differed in that the balloon was deflated by puncturing the coronary artery with a thin needle, eliminating the need for a coronary artery incision. This approach is expected to reduce surgical time and minimize patient burden.

In this case, repeated punctures and external compression of the coronary segment were necessary to expel the contrast from the balloon. This was caused by the tight compression of the balloon against the coronary artery wall and stent, limiting passive deflation via a single puncture. A larger needle could have created a sufficient escape path for deflation, but it poses the risk of complications such as bleeding or vessel injury. Although the optimal needle size remains unclear, the use of a thin 25-G needle under direct visualization minimizes these risks. No bleeding or vessel injury was observed during or after the procedures.

Performing off-pump surgery and retrieving the balloon without coronary incision through a small thoracotomy reduced the invasiveness of the procedure and resulted in the rescue of our patient with cardiogenic shock. Interventional cardiologists can change the retrieval methods from endovascular to surgical techniques when the balloon shaft remains intact, enabling successful retrieval through the catheter after deflation. Given the life-threatening nature of this complication, interventional cardiologists should be familiar with its management.

## Conclusions

An undeflatable balloon is a rare but potentially fatal PCI complication that may require invasive retrieval if catheter-based methods fail. In such cases, a minimally invasive surgical approach including off-pump surgery and direct needle puncture with a small thoracotomy may be a viable alternative to coronary artery incision, particularly in patients with elevated surgical risk.

## Funding Support and Author Disclosures

The authors have reported that they have no relationships relevant to the contents of this paper to disclose.

## Appendix

Video 1 Undeflatable Balloon in Coronary Artery Coronary angiography showing an undeflatable balloon lodged in the mid-left anterior descending artery. Despite forceful traction, only the guiding catheter was drawn inward, and the balloon remained entrapped.

Video 2 Needle Puncture of an Undeflatable Balloon A 25-G needle was used to puncture the balloon from the coronary artery surface. Despite multiple attempts, deflation was not achieved.

Video 3 Squeezing the Punctured Balloon Using Forceps After repeated punctures, the balloon was manually squeezed with forceps, resulting in complete deflation.

Video 4 Retrieval of the Deflated Balloon The deflated balloon was retrieved via the guiding catheter without requiring coronary artery incision.

Video 5 Final Coronary Angiography Final angiography confirmed restored coronary flow without evidence of extravasation.

Supplemental Figure 1 Pre-procedural Coronary Angiography Showing Intermediate Stenosis in the Mid-LAD.
