# Supplementary material for: Retrieval of an Undeflatable Balloon During Percutaneous Coronary Intervention in Combination With Small Thoracotomy
Source: JACC Case Rep. 2025 Sep 24;30(29):105157. doi: 10.1016/j.jaccas.2025.105157 (PMC12539485; doi:10.1016/j.jaccas.2025.105157)
Supplement: Supplemental Figure 1 — Pre-procedural Coronary Angiography Showing Intermediate Stenosis in the Mid-LAD. [file mmc6.docx]

**Figure S1**

**
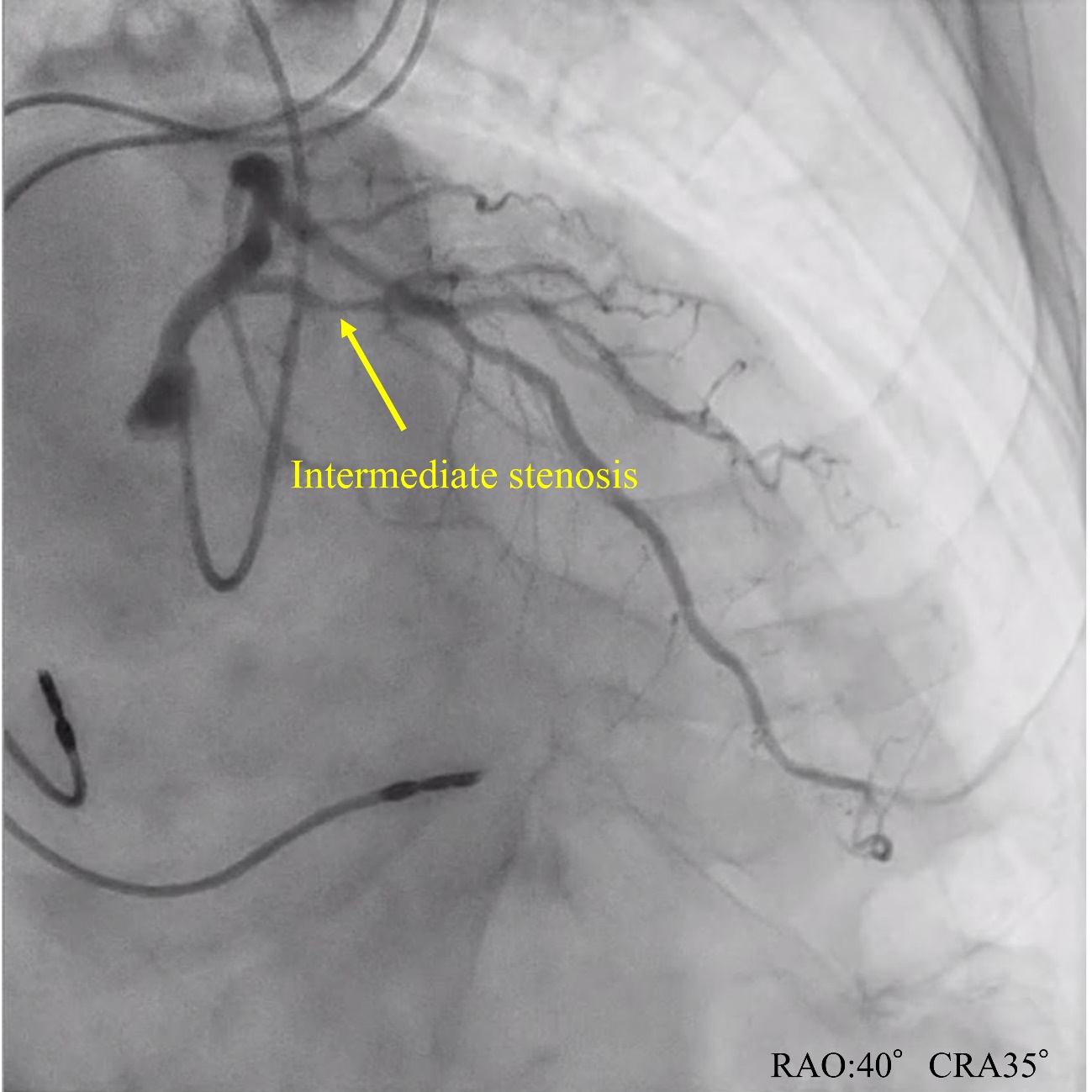
**

**Figure S1**. Pre-procedural coronary angiography showing intermediate stenosis in the mid left anterior descending artery.

Coronary angiography demonstrating intermediate stenosis in the mid left anterior descending artery.

**Equipment List**

**Imaging**

- Fluoroscopy machine, *Philips Healthcare*, Andover, MA, USA

**Access**

- Radifocus® Introducer II H, 6 Fr, 16 cm, *Terumo Corporation*, Tokyo, Japan
- Radifocus® Introducer II H, 7 Fr, 50 cm, *Terumo Corporation*, Tokyo, Japan
- Radifocus® Introducer II H, 8 Fr, 10 cm, *Terumo Corporation*, Tokyo, Japan

**Percutaneous Coronary Intervention**

- Taiga™ SL35, 6 Fr, *Medtronic, Inc.*, Minneapolis, MN, USA
- Taiga™ JL40, 6 Fr, *Medtronic, Inc.*, Minneapolis, MN, USA
- GUIDEPLUS™ II, 6 Fr, *Nipro Corporation*, Osaka, Japan
- Runthrough™ NS Ultra Floppy, *Terumo Corporation*, Tokyo, Japan
- WOLVERINE™ Cutting Balloon, *Boston Scientific*, Marlborough, MA, USA
- XIENCE Skypoint™, *Abbott Vascular*, Santa Clara, CA, USA
- NC Euphora™, *Medtronic, Inc.*, Minneapolis, MN, USA
- Hiryu™ Plus, *Terumo Corporation*, Tokyo, Japan

**Catheter-Based Retrieval**

- Heartrail™ II ST01, *Terumo Corporation*, Tokyo, Japan
- KIWAMI™ ST01, *Terumo Corporation*, Tokyo, Japan
- GUIDEZILLA™ II Guide Extension Catheter, *Boston Scientific*, Marlborough, MA, USA
- Taiga™ JL40, 6 Fr, *Medtronic, Inc.*, Minneapolis, MN, USA
- X-treme™ XT-R, *Asahi Intecc Co., Ltd.*, Aichi, Japan
- SION™ Black, *Asahi Intecc Co., Ltd.*, Aichi, Japan
- Gaia™ Next 1, *Asahi Intecc Co., Ltd.*, Aichi, Japan
- Conquest Pro™ 12 ST, *Asahi Intecc Co., Ltd.*, Aichi, Japan
- TAU™ Intra-Aortic Balloon Pump, 7 Fr, 35 cc, 205 mm, *Tokai Medical Products*, Aichi, Japan

**Surgical Retrieval**

- Standard cardiovascular surgical instrument set
- 1 mL tuberculin syringe with 25G needle, *Terumo Corporation*, Tokyo, Japan
